# Endoplasmic Reticulum Stress Disturbs Lipid Homeostasis and Augments Inflammation in the Intestine and Isolated Intestinal Cells of Large Yellow Croaker (*Larimichthys crocea*)

**DOI:** 10.3389/fimmu.2021.738143

**Published:** 2021-08-19

**Authors:** Wei Fang, Qiuchi Chen, Jiamin Li, Yongtao Liu, Zengqi Zhao, Yanan Shen, Kangsen Mai, Qinghui Ai

**Affiliations:** ^1^Key Laboratory of Aquaculture Nutrition and Feed (Ministry of Agriculture and Rural Affairs) and Key Laboratory of Mariculture (Ministry of Education), Ocean University of China, Qingdao, China; ^2^Laboratory for Marine Fisheries Science and Food Production Processes, Qingdao National Laboratory for Marine Science and Technology, Qingdao, China

**Keywords:** unfolded protein response, tunicamycin, lipid metabolism, inflammatory response, intestine

## Abstract

The small intestine is crucial for lipid homeostasis and immune regulation of the whole body. Endoplasmic reticulum (ER) stress may affect lipid metabolism and inflammation in the intestine, but the potential mechanism is not completely understood. In the present study, intraperitoneal injection of tunicamycin (TM) induced ER stress in the intestine of large yellow croaker (*Larimichthys crocea*). ER stress induced excessive accumulation of triglyceride (TG) in the intestine by promoting lipid synthesis. However, it also enhanced lipid secretion and fatty acid β-oxidation. In addition, ER stress augmented inflammation in the intestine by promoting p65 into the nucleus and increasing proinflammatory genes expression. In the isolated intestinal cells, the obtained results showed that TM treatment significantly upregulated the mRNA expression of lipid synthesis and inflammatory response genes, which were consistent with those *in vivo*. Moreover, overexpression of unfolded protein response (UPR) sensors significantly upregulated promoter activities of lipid synthesis and proinflammatory genes. In conclusion, the results suggested that ER stress disturbed lipid metabolism and augmented inflammation in the intestine and isolated intestinal cells of large yellow croaker, which may contribute to finding novel therapies to tackle lipid dysregulation and inflammation in the intestine of fish and human beings.

## Introduction

The small intestine is abundant in intestinal microvilli composed of enterocytes, goblet cells, and enteroendocrine cells (1). Intestine plays an important role in digesting and absorbing exogenous lipids and supplying adequate energy (in the form of lipids) to the various organs in the body, which critically contributes to maintaining the whole-body lipid homeostasis (2). The lipid metabolism in the intestine is complicated, including uptake of lipids, lipid synthesis, chylomicrons secretion, cytoplasmic lipid droplets storage, and fatty acid β-oxidation (3). Lipid dysregulation in the intestine may be associated with various adverse health conditions (3, 4).

The endoplasmic reticulum (ER) is important for regulating protein folding, macromolecule biosynthesis, and calcium storage. Millions of proteins synthesize in the ER. However, not all of them can be correctly folded. Various unfolded or misfolded proteins accumulating in the lumen of ER cause ER stress and activate unfolded protein response (UPR), including the inositol-requiring protein 1 α (IRE1α) pathway, the transcription factor 6 (ATF6) pathway, and the double-stranded RNA-dependent protein kinase-like ER kinase (PERK) pathway. Several enzymes that are involved in lipid metabolism locate in the ER (5). The UPR sensors have been proved to play a crucial role in regulating lipid metabolism (6). For example, hepatic-specific deletion of IRE1α increased the peroxisome proliferator-activated receptor γ (PPARγ) level to increase the hepatic lipid content (7). The activation of the PERK pathway increased lipid accumulation in cells by activating sterol regulatory element binding protein 1c (SREBP-1c) (8). Chen et al. (9) found that ATF6 increases fatty acid β-oxidation through peroxisome proliferator-activated receptor α (PPARα). Although numerous studies have investigated the mechanism of ER stress affecting hepatic lipid homeostasis, the mechanism of ER stress-mediated intestinal lipid metabolism is not completely understood.

In addition, evidence is accumulating that ER stress and activation of the UPR pathway may be the primary contributors to the development of chronic inflammation in the intestine (10, 11). The activation of IRE1α recruits tumor necrosis factor receptor 1 (TNFR1) to increase proinflammatory gene expression by activating MAP kinase c-Jun NH2-terminal kinase (JNK) (12). PERK mediates apoptosis and promotes proinflammatory gene expression through activating transcription factor 4 (ATF4) (13, 14). Upregulating the C/EBP homologous protein (CHOP) expression could exacerbate the development of colitis (15). Despite the fact that these findings were certified, the underlying mechanism of ER stress and the UPR pathway on inflammation in the intestine remains unclear and needs further study.

Although fish are less evolutionary than mammals, the nutrient- and pathogen-sensing and immune response were evolutionarily conserved (16). They are susceptible and vulnerable to nutritional stress and aquatic environments, which leads to ER stress, lipid dysregulation, and inflammation in the intestine of fish (17–19). Therefore, fish is a good model to research the pathogenesis of metabolic disease and inflammation in the intestine. This study aims to investigate how ER stress mediates lipid metabolism and inflammation in the intestine of fish, which may contribute to finding novel therapies to prevent and treat lipid dysregulation and inflammation in the intestine of fish and human beings.

## Materials and Methods

### Animal Experiment

Large yellow croaker juveniles were bought from a commercial fish farm (Ningbo, China). After 1 week of acclimation, the fish were randomly divided into two groups equally, which were respectively injected with dimethyl sulfoxide (DMSO) (Solarbio, China) or Tunicamycin (TM) (Sigma, USA) (3 μg/g fish). Fish were anesthetized with MS-222 and collected at 24 h after injection. The intestines of the fish were immediately collected and frozen in liquid nitrogen, and then stored at -80°C for further analysis.

### Culture and Treatment of Intestinal Cells

Intestinal cells were isolated from healthy large yellow croaker and cultured in Dulbecco’s Modified Eagle Medium/Ham’s F12 (DMEM/F12) medium (Biological Industries, Israel) with 15% fetal bovine serum (FBS) (Biological Industries) and penicillin and streptomycin (Solarbio) in 5% CO_2_ atmosphere at 27°C. Intestinal cells were seeded into 6-well plates and cultured overnight. Cells were treated with 1 μM TM for different time points to explore the effects of ER stress on intestinal lipid metabolism and inflammation *in vitro*. Then cells were harvested for further analysis.

### TG Content and Lipid Droplet Staining

The TG content was analyzed according to a previous study (20). After TM treatment for 12 h, cells were incubated with BODIPY 493/503 (Sigma) for 10 min, washed with phosphate buffer solution (PBS, Solarbio) three times, and then immediately observed lipid droplets in the cells with a fluorescent microscope (Nikon, Japan), according to the methods in a previous study (21).

### RNA Isolation and Quantitative Real-Time Polymerase Chain Reaction (qRT-PCR)

The total RNA of the intestines and isolated intestinal cells was prepared using the TRIzol reagent (Takara, Japan), and cDNA was synthesized using the PrimeScript™ RT reagent kit (Takara), according to the method in our previous study (22). qRT-PCR was performed using SYBR Premix Ex Taq (Takara) and Roche LightCycler^®^ 96 system (USA). The specific primer sequences of genes were designed according to published sequences in NCBI and listed in **Table 1**. In the present study, we found that the most stable reference gene was *βactin* across all the samples rather than 18S rRNA or *gapdh*, according to geNorm analysis (23). Thus, *βactin* was used as a reference gene. The relative mRNA expression of related genes was calculated using the comparative CT method (24).

**Table 1:** Primer sequences of genes used for quantitative real-time PCR.

Sequence Name	Forward 5’-3’	Reverse 5’-3’
*grp78*	GGTGGCGATGACAAGCAAAC	CTGAGAACAGCAGCAACAAGC
*xbp1*	GTCTTCTGAGTCCGCAGCAGGTG	AGGATGTCCAGAATGCCCAGTAG
*atf4*	GCCGTTATTCTGCTCCATCTTCT	AGACCTTACCCTGAGCCCACAT
*atf6*	CAGATAATAAGGAGGCTGAGAGTGC	CGTAGGTATGATGAGGTGCGTAGT
*chop*	TCTGGATGTTCTGGAGAGTTGTTC	AGGATGATGATGAGGTGTGATGC
*cd36*	CAGGCAGTTCTGGTTATTTGATTTG	GCAGCAGGAAGGAGACAGTGTTATT
*fatp1*	CAACCAGCAGGACCCATTACG	CATCCATCACCAGCACATCACC
*fatp4*	TCAACGACCGAGGTGGAGGG	CGGAAGGAAGCGGAGGAACA
*fabp1*	AGGCTATTGGTCTTCCTGATGA	AGGACCTTAGTGCCAGTAGTGA
*fabp2*	GGGTCACCTTTGAGTACAGCCTTG	CCTTCTTGAAAATCCTCTTTGCGT
*fabp3*	CCAAACCCACCACTATCATCTCAG	GCACCATCTTTCCCTCCTCTATTG
*srebp1c*	TCTCCTTGCAGTCTGAGCCAAC	TCAGCCCTTGGATATGAGCCT
*scd1*	AAAGGACGCAAGCTGGAACT	CTGGGACGAAGTACGACACC
*acc1*	GACTTGGCGGAATACCTACTGG	GCTTGCTGGATGATCTTTGCTT
*acc2*	AAAGAATCCCTGTGCAGGCTGTC	TCCTCCTCGGTCCAATCCACTC
*dgat1*	GGTATCTTGGTGGACCCCATTCA	TGAGCACCGTGGCTGAAGGAAAGA
*dgat2*	TTCGGTGCTTTCTGCAACTTCG	AAGGATGGGGAAGCGGAAGT
*adrp*	CAAGGCTAATGCGTTGGAAGA	AGTTGAGCGGCGTGTTATTGA
*pparα*	GTCAAGCAGATCCACGAAGCC	TGGTCTTTCCAGTGAGTATGAGCC
*cpt1α*	GCTGAGCCTGGTGAAGATGTTC	TCCATTTGGTTGAATTGTTTACTGTCC
*aco*	AGTGCCCAGATGATCTTGAAGC	CTGCCAGAGGTAACCATTTCCT
*mtp*	CTTGAGTCGCTGATTGCTGC	TGAGGTCGCTGTAACCCTTG
*apob*	AGAGTGTTGTCCAGGATAAAGATGC	CAGGGCTCAGGGTCTCAGTC
*sar1b*	GCATGACTTTCACCACCTTTG	GTTCTGCTTTTGATTCTCCCA
*sec13*	CTCCTTCTATTGGTCTCCCC	ACAGCGTCACCTTGTTGTCT
*sec31*	CTGGTGGAGAAGGTGGTGGT	GTGTTGTCGGGCAGGTAGGT
*sec23*	ACACCAGTCATACCTACCGC	AGATCCTCAAACTCTTCCCC
*sec24*	TCCCCAGCGACAGATTTCTA	TTGGTGCAGCGTATCCTCAT
*il-1β*	CATAGGGATGGGGACAACGA	AGGGGACGGACACAAGGGTA
*il-6*	CGACACACCCACTATTTACAAC	TCCCATTTTCTGAACTGCCTCT
*il-8*	AATCTTCGTCGCCTCCATTGT	GAGGGATGATCTCCACCTTCG
*cox2*	CTGGAAAGGCAACACAAGC	CGGTGAGAGTCAGGGACAT
*tnfα*	ACACCTCTCAGCCACAGGAT	CCGTGTCCCACTCCATAGTT
*βactin*	GACCTGACAGACTACCTCATG	AGTTGAAGGTGGTCTCGTGGA

grp78, glucose related protein 78; xbp1, X-box binding protein 1; atf4, activating transcription factor 4; atf6, activating transcription factor 6; chop, C/EBP homologous protein; cd36, fatty acid translocase; fatp1, fatty acid transport protein 1; fatp4, fatty acid transport protein 4; fabp1, fatty acid-binding protein 1; fabp2, fatty acid-binding protein 2; fabp3, fatty acid-binding protein 3; srebp1c, sterol regulatory element binding protein 1 c; scd1, stearoyl-CoA desaturase 1; acc1, acetyl-CoA carboxylase 1; acc2, acetyl-CoA carboxylase 2; dgat1, diacylglycerol acyltransferase 1; dgat2, diacylglycerol acyltransferase 2; adrp, adipose differentiation-related protein; pparα, peroxisome proliferator-activated receptor alpha; cpt1α, carnitine palmitoyl transferase 1 alpha; aco, acyl-CoA oxidase; mtp, microsomal triglyceride transfer protein; apob, apolipoprotein; sar1b, secretion associated Ras related GTPase 1B; sec13, sec13 homolog, nuclear pore and COPII coat complex component; sec31, sec31 homolog A, COPII coat complex component; sec23, sec23 homolog A, coat complex II component; sec24, sec24 homolog A, COPII coat complex component; il-1β, interleukin-1 beta; il-6, interleukin-6; il-8, interleukin-8; cox2, cyclooxygenase 2; tnfα, tumor necrosis factor alpha; βactin, Beta-actin.

### Western Blotting Analysis

The total protein of the sample was extracted by adding RIPA lysis (Solarbio), and nuclear protein was collected using NE-PER Nuclear and Cytoplasmic Extraction Reagents (Thermo Fisher Scientific, USA), according to the method in our previous study (25). The protein concentration was measured by a BCA Protein Assay Kit (Beyotime, China) for adjustments. The protein was separated on 10% sodium dodecyl sulfate polyacrylamide gel electrophoresis (SDS-PAGE) at 150 V for 1 h and transferred to 0.45 μm polyvinylidene fluoride (PVDF) membranes (Millipore, USA) at 100 V for 1 h. Then the PVDF membranes were blocked with 5% skimmed milk at room temperature for 1 h, followed by overnight incubation at 4°C with specific primary antibodies, including glucose-regulated protein 78 (GRP78) (3117, CST, USA), X-box-binding protein 1 s (XBP1s) (12782, CST), ATF6 (bs-1634R, Bioss, China), phosphorylated double-stranded RNA-dependent protein kinase-like ER kinase (p-PERK) (Thr982) (137056, Absin, China), acetyl-CoA carboxylase (ACC) (3662, CST), diacylglycerol acyltransferase 1 (DGAT1) (Gensript, China), cleavage of sterol-regulatory element binding protein 1 c (SREBP1c) (WL02093, Wanleibio, China), fatty acid translocase (FAT/CD36) (Gensript), microsomal triglyceride transfer protein (MTP) (ab186446, Abcam, England), apolipoprotein B 48 (APOB48) (Gensript), GTPase-activating protein SEC13 (SEC13) (sc-514308, Santa Cruz, USA), secretion associated Ras related GTPase 1B (SAR1B) (ab155278, Abcam), acyl-CoA oxidase (ACO) (ab184032,Abcam), carnitine palmitoyl transferase 1 α (CPT1α) (15184-1-AP, Proteintech, USA), peroxisome proliferator-activated receptor-γ coactlvator-1α (PGC1α) (ab118102, Abcam), peroxisome proliferator-activated receptor α (PPARα) (117362, Absin), extracellular-regulated kinase 1/2 (ERK1/2) (4695, CST), p-ERK1/2 (Thr202/Tyr204) (4370, CST), p38 mitogen-activated protein kinase (p38) (8690, CST), p-p38 (Thr180/Tyr182) (9215, CST), NF-kappaB p65 (p65) (8242, CST), interleukin-1 β (IL-1β) (Gensript), glyceraldehyde-3-phosphate dehydrogenase (GAPDH) (R001, Goodhere, China), and Histone H3 (ab1791, Abcam). Then the protein bands were incubated with HRP-conjugated secondary antibody (A0208, Beyotime) for 1 h at room temperature. The immunoreactive protein was detected using ELC reagent (Beyotime).

### Plasmid Construction and Dual-Luciferase Reporter Assays

The *acc*, *scd1*, *dgat1*, *dgat2*, *il-1β*, *il-6*, *tnfα*, and *cox2* promoter fragments of large yellow croaker were cloned, and then they were constructed into the PGL3-basic vector to assemble reporter plasmids, respectively, using a ClonExpress II One Step Cloning Kit (Vazyme Biotech, China), according to previously described methods (26). The XBP1, CHOP, ATF4, and ATF6 CDS fragments were cloned and constructed into a PCS2+ vector to assemble expression plasmids, respectively. According to the manufacturer’s instructions, all plasmids for transfection were prepared by using the EasyPure HiPure Plasimid MinPrep Kit (TransGen Biotech, China).

HEK293T cells were cultured in DMEM high glucose medium (Biological Industries) with 10% FBS (Biological Industries) and penicillin and streptomycin (Solarbio) in 5% CO_2_ atmosphere at 37°C. To determine the effects of UPR sensors on the promoter activities of lipid synthesis and proinflammatory genes, HEK293T cells were cotransfected with reporter plasmid, expression plasmid, and phRL-CMV plasmid using Lipofectamine 2000 (Invitrogen, USA). After transfection for 24 h, cells were harvested and measured for luciferase activity using a Dual-Luciferase Reporter Assay Kit (TransGen Biotech), according to the manufacturer’s instructions.

### Data Analysis

Statistical evaluations were analyzed with independent sample *t*-test or one-way analysis of variance followed by Tukey’s multiple-range test. The analysis was carried out using the SPSS 17.0 software (IBM, USA). The results were presented as mean ± standard deviation. *P* < 0.05 was considered statistically significant.

## Results

### TM Injection Induces ER Stress in the Intestine of Large Yellow Croaker *In Vivo*

**Figure 1:**
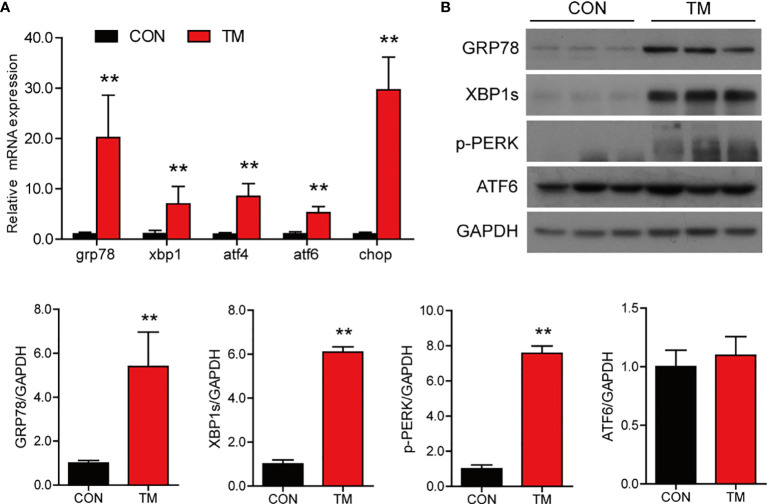
TM injection induces ER stress in the intestine of large yellow croaker *in vivo*. **(A)** Relative mRNA expression of ER stress-related genes in the intestine of large yellow croaker after TM injection for 24 h (n = 6). **(B)** Western blot analysis of GRP78, XBP1s, p-PERK, and ATF6 protein levels in the intestine of large yellow croaker after TM injection for 24 h (normalized to GAPDH, n = 3). *grp78*, glucose related protein 78; *xbp1*, X-box binding protein 1; *atf4*, activating transcription factor 4; *atf6*, activating transcription factor 6; *chop*, C/EBP homologous protein; PERK, double-stranded RNA-dependent protein kinase-like ER kinase; GAPDH, glyceraldehyde-3-phosphate dehydrogenase. Results were analyzed using independent *t*-test (**P < 0.01), and they were presented as mean ± standard deviation.

To investigate the role of ER stress in the regulation of lipid homeostasis and inflammation in the intestine, we injected TM into large yellow croaker. Compared with the control group, the mRNA expression of ER stress-related genes including *grp78*, *xbp1s*, *atf4*, *atf6*, and *chop* was significantly increased in the intestine after TM injection (*P* < 0.05) (**Figure 1A**). Besides, the protein levels of GRP78, XBP1s, and p-PERK were significantly upregulated (*P* < 0.05), while the protein level of ATF6 was not significantly changed in the TM group (*P* > 0.05) (**Figure 1B**). These results indicated that TM injection induced ER stress in the intestine of large yellow croaker *in vivo*.

### ER Stress Disrupts Lipid Metabolism Homeostasis in the Intestine of Large Yellow Croaker *In Vivo*

**Figure 2:**
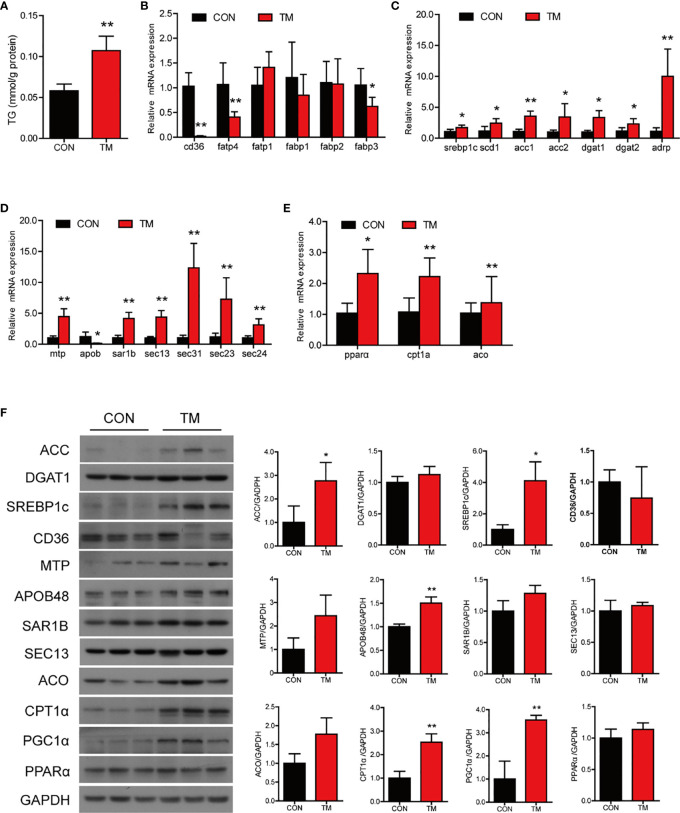
ER stress disrupts lipid metabolism homeostasis in the intestine of large yellow croaker *in vivo*. **(A)** TG content in the intestine of large yellow croaker after TM injection for 24 h (n = 6). Relative mRNA expression of fatty acid uptake **(B)**, lipid synthesis **(C)**, chylomicron secretion **(D),** and fatty acid β-oxidation **(E)** in the intestine of large yellow croaker after TM injection for 24 h (n = 6). **(F)** Western blot analysis of lipid metabolism related protein levels in the intestine of large yellow croaker after TM injection for 24 h (normalized to GAPDH, n = 3). *cd36*, fatty acid translocase; *fatp1*, fatty acid transport protein 1; *fatp4*, fatty acid transport protein 4; *fabp1*, fatty acid-binding protein 1; *fabp2*, fatty acid-binding protein 2; *fabp3*, fatty acid-binding protein 3; *srebp1c*, sterol regulatory element binding protein 1 c; *scd1*, stearoyl-CoA desaturase 1; *acc1*, acetyl-CoA carboxylase 1; *acc2*, acetyl-CoA carboxylase 2; *dgat1*, diacylglycerol acyltransferase 1; *dgat2*, diacylglycerol acyltransferase 2; *adrp*, adipose differentiation-related protein; *mtp*, microsomal triglyceride transfer protein; *apob*, apolipoprotein; *sar1b*, secretion associated Ras related GTPase 1B; *sec13*, sec13 homolog, nuclear pore and COPII coat complex component; *sec31*, sec31 homolog A, COPII coat complex component; *sec23*, sec23 homolog A, coat complex II component; *sec24*, sec24 homolog A, COPII coat complex component; *pparα*, peroxisome proliferator-activated receptor alpha; *cpt1α*, carnitine palmitoyl transferase 1 alpha; *aco*, acyl-CoA oxidase; PGC1α, peroxisome proliferator-activated receptor gamma coactivator 1alpha; GAPDH, glyceraldehyde-3-phosphate dehydrogenase. Results were analyzed using independent *t*-test (*P < 0.05, **P < 0.01), and they were presented as mean ± standard deviation.

Compared with the control group, TM injection significantly increased the TG content in the intestine (*P* < 0.05) (**Figure 2A**). Lipid homeostasis in the intestine is maintained through multiple pathways, including fatty acid uptake, lipid synthesis, lipid secretion, and fatty acid β-oxidation. In terms of lipid uptake, the results showed that the mRNA levels of *cd36*, *fatp4*, and *fabp3* in the intestine were significantly downregulated in the TM group (*P* < 0.05) (**Figure 2B**). TM injection significantly increased the mRNA expression of genes related lipid synthesis, including *srebp1c*, *scd1*, *acc1*, *acc2*, *dgat1*, *dgat2*, and *adrp* (*P* < 0.05) (**Figure 2C**). Compared with the control group, TM injection significantly increased mRNA levels of *mtp*, *sar1b*, *sec13*, *sec31*, *sec23*, and *sec24* (*P* < 0.05), while the m RNA level of *apob* was significantly decreased in the TM group (*P* < 0.05) (**Figure 2D**). We also found that the mRNA levels of *pparα*, *cpt1α*, and *aco* were increased after TM injection (*P* < 0.05) (**Figure 2E**). Compared with the control group, the protein levels of ACC, SREBP1c, APOB48, CPT1α, and PGC1α were significantly upregulated (*P* < 0.05) (**Figure 2F**). Collectively, these results indicated that ER stress disturbed lipid metabolism leading to abnormal lipid accumulation in the intestine of large yellow croaker.

### ER Stress Augments Inflammatory Response in the Intestine of Large Yellow Croaker *In Vivo*

**Figure 3:**
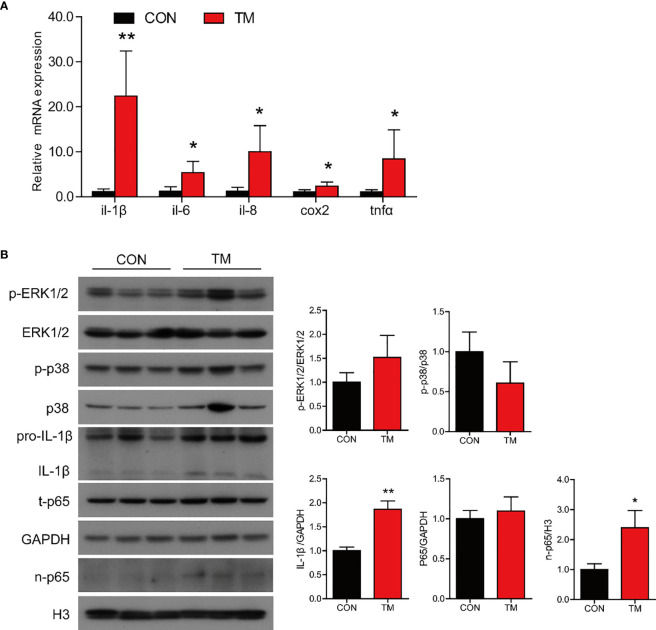
ER stress augments inflammatory response in the intestine of large yellow croaker *in vivo*. **(A)** Relative mRNA expression of inflammation-related genes in the intestine of large yellow croaker after TM injection for 24 h (n = 6). **(B)** Western blot analysis of inflammation-related protein levels in the intestine of large yellow croaker after TM injection for 24 h (normalized to GAPDH, n = 3). *il-1β*, interleukin-1 beta; *il-6*, interleukin-6; *il-8*, interleukin-8; *cox2*, cyclooxygenase 2; *tnfα*, tumor necrosis factor alpha; ERK1/2, extracellular signal-regulated protein kinases 1 and 2; p38, p38 mitogen-activated protein kinase; p65, NF-kappaB p65; GAPDH, glyceraldehyde-3-phosphate dehydrogenase. Results were analyzed using independent *t*-test (*P < 0.05, **P < 0.01), and they were presented as mean ± standard deviation.

TM injection significantly promoted mRNA expression of proinflammatory genes in the intestine, including *il-1β*, *il-6*, *il-8*, *cox2*, and *tnfα* (*P* < 0.05) compared with the control group (**Figure 3A**). Compared with the control group, the protein levels of IL-1β and nuclear p65 were significantly increased (*P* < 0.05), while the protein levels of p-ERK1/2/ERK1/2, p-p38/p38, and total p65 were not remarkably different in the TM group (*P* > 0.05) (**Figure 3B**). The results above indicated that ER stress augmented inflammatory response in the intestine of large yellow croaker.

### TM Treatment Induces ER Stress in the Isolated Intestinal Cells of Large Yellow Croaker *In Vitro*

**Figure 4:**
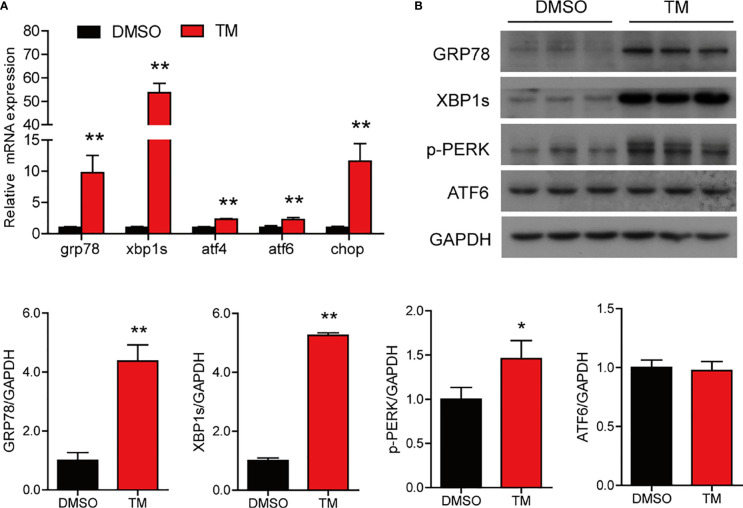
TM treatment induces ER stress in the isolated intestinal cells of large yellow croaker *in vitro*. **(A)** Relative mRNA expression of ER stress-related genes in the isolated intestinal cells of large yellow croaker after TM treatment for 6 h (n = 3). **(B)** Western blot analysis of GRP78, XBP1s, p-PERK, and ATF6 protein levels in the isolated intestinal cells of large yellow croaker after TM treatment for 6 h (normalized to GAPDH, n = 3). *grp78*, glucose related protein 78; *xbp1*, X-box binding protein 1; *atf4*, activating transcription factor 4; *atf6*, activating transcription factor 6; *chop*, C/EBP homologous protein; PERK, double-stranded RNA-dependent protein kinase-like ER kinase; GAPDH, glyceraldehyde-3-phosphate dehydrogenase. Results were analyzed using independent *t*-test (*P < 0.05, **P < 0.01), and they were presented as mean ± standard deviation.

To further investigate that ER stress induced by TM mediated lipid metabolism and inflammation in the intestine of large yellow croaker, the intestinal cells were isolated from large yellow croaker and incubated with 1 μM TM at different time points. The results showed that TM treatment significantly increased the mRNA levels of *grp78*, *xbp1s*, *atf4*, *atf6*, and *chop* compared with the control group (*P* < 0.05) (**Figure 4A**). Meanwhile, the protein levels of GRP78, XBP1s, and p-PERK were significantly higher than those in the control group (*P* < 0.05), while the protein level of ATF6 was not remarkably different (*P* > 0.05) (**Figure 4B**). The results suggested that TM incubation could induce ER stress in the isolated intestinal cells of large yellow croaker *in vitro*.

### ER Stress Disrupts Lipid Metabolism Homeostasis in the Isolated Intestinal Cells of Large Yellow Croaker *In Vitro*

**Figure 5:**
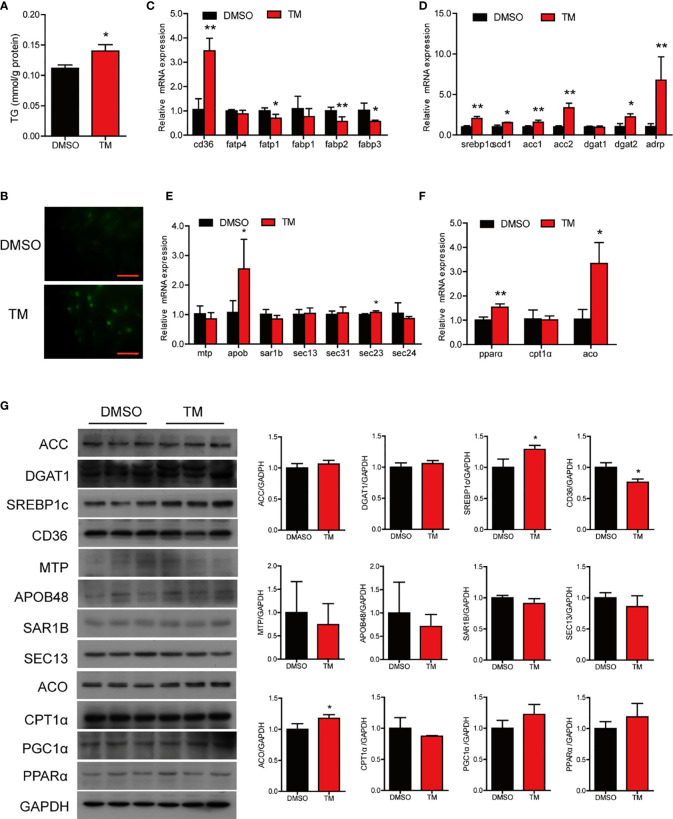
ER stress disrupted lipid metabolism homeostasis in the isolated intestinal cells of large yellow croaker *in vitro*. **(A)** TG content in the intestinal cells after TM treatment for 12 h (n = 3). **(B)** Lipid droplets in cells stained with BODIPY 493/503 (magnification: 200×, scale bars: 60 μm). Relative mRNA expression of fatty acid uptake **(C)**, lipid synthesis **(D)**, chylomicron secretion **(E),** and fatty acid β-oxidation **(F)** in the isolated intestinal cells of large yellow croaker after TM treatment for 12 h (n = 3). **(G)** Western blot analysis of lipid metabolism related protein levels in the isolated intestinal cells of large yellow croaker after TM treatment for 12 h (normalized to GAPDH, n = 3). *cd36*, fatty acid translocase; *fatp1*, fatty acid transport protein 1; *fatp4*, fatty acid transport protein 4; *fabp1*, fatty acid-binding protein 1; *fabp2*, fatty acid-binding protein 2; *fabp3*, fatty acid-binding protein 3; *srebp1c*, sterol regulatory element binding protein 1 c; *scd1*, stearoyl-CoA desaturase 1; *acc1*, acetyl-CoA carboxylase 1; *acc2*, acetyl-CoA carboxylase 2; *dgat1*, diacylglycerol acyltransferase 1; *dgat2*, diacylglycerol acyltransferase 2; *adrp*, adipose differentiation-related protein; *mtp*, microsomal triglyceride transfer protein; *apob*, apolipoprotein; *sar1b*, secretion associated Ras related GTPase 1B; *sec13*, sec13 homolog, nuclear pore and COPII coat complex component; *sec31*, sec31 homolog A, COPII coat complex component; *sec23*, sec23 homolog A, coat complex II component; *sec24*, sec24 homolog A, COPII coat complex component; *pparα*, peroxisome proliferator-activated receptor alpha; *cpt1α*, carnitine palmitoyl transferase 1 alpha; *aco*, acyl-CoA oxidase; PGC1α, peroxisome proliferator-activated receptor gamma coactivator 1alpha; GAPDH, glyceraldehyde-3-phosphate dehydrogenase. Results were analyzed using independent *t*-test (*P < 0.05, **P < 0.01), and they were presented as mean ± standard deviation.

We next examined the effect of ER stress on lipid metabolism in the intestinal cells. Compared with the control group, TM treatment significantly increased the level of TG (*P* < 0.05) (**Figure 5A**) and the number and size of lipid droplets in cells (**Figure 5B**). In terms of lipid uptake, TM treatment significantly increased the mRNA level of *cd36* (*P* < 0.05) and decreased the mRNA levels of *fatp1*, *fabp2*, and *fabp3* (*P* < 0.05) (**Figure 5C**). The mRNA levels of lipid synthesis, including *srebp1c*, *scd1*, *acc1*, *acc2*, *dgat2*, and *adrp*, were significantly increased after TM treatment (*P* < 0.05) (**Figure 5D**). Compared with the control group, TM treatment significantly upregulated the gene levels of *apob* and *sec23*, while the mRNA levels of *mtp*, *sar1b*, *sec13*, *sec31*, *sec23*, and *sec24* were not remarkably different (*P* > 0.05) (**Figure 5E**). For fatty acid β-oxidation, the gene levels of *pparα* and *aco* were significantly upregulated (*P* < 0.05) (**Figure 5F**). Western bolt results showed that the protein levels of SREBP1c and ACO were significantly upregulated, and the protein level of CD36 was downregulated (*P* < 0.05) (**Figure 5G**). In agreement with TM injection *in vivo*, these results indicated that ER stress boosted the lipid synthesis leading to abnormal TG accumulation in cells.

### UPR Sensors Regulate Promoter Activities of Lipid Synthesis Related Genes

**Figure 6:**
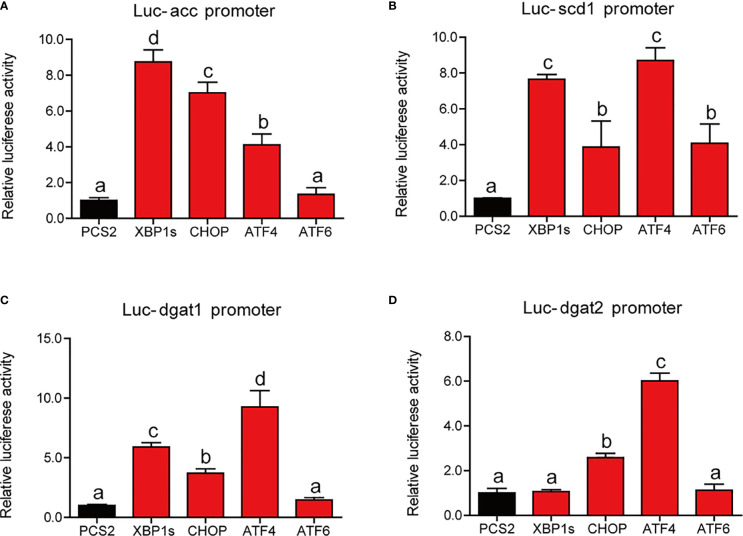
Relative dual-luciferase activity analysis of UPR sensors in promoters of lipid synthesis-related gene in HEK293T cells. The promoter activities of *acc* **(A)**, *scd1* **(B)**, *dgat1* **(C)**, and *dgat2* **(D)** were measured (n = 4). *acc*, acetyl-CoA carboxylase; *scd1*, stearoyl-CoA desaturase 1; *dgat1*, diacylglycerol acyltransferase 1; *dgat2*, diacylglycerol acyltransferase 2. Results were analyzed using Tukey’s test [values without the same letter indicate significant difference (*P* < 0.05)], and they were expressed as mean ± standard deviation.

We have established that ER stress disrupted lipid metabolism in the intestine and isolated the intestinal cells of large yellow croaker. To further investigate the regulatory mechanism, we cotransfected the reporter plasmids of lipid synthesis promoters and expression plasmids of UPR sensors into HEK293T cells. Compared with HEK293T cells transfected with PCS2+ plasmid, cells transfected with XBP1s, CHOP, or ATF4 expression plasmid showed significantly higher luciferase activity of the *acc* promoter of large yellow croaker (*P* < 0.05) (**Figure 6A**). Similarly, the promoter activity of *scd1* in cells that transfected with UPR sensor plasmid was significantly higher than those transfected with PCS2+ plasmid (*P* < 0.05) (**Figure 6B**). Overexpression of XBP1s, CHOP, or ATF4 significantly promoted the promoter activity of *dgat1* (*P* < 0.05) (**Figure 6C**). Overexpression of CHOP or ATF4 significantly upregulated the promoter activity of *dgat2* compared with the control group (*P* < 0.05) (**Figure 6D**).

### ER Stress Augments Inflammatory Response in the Isolated Intestinal Cells of Large Yellow Croaker *In Vitro*

**Figure 7:**
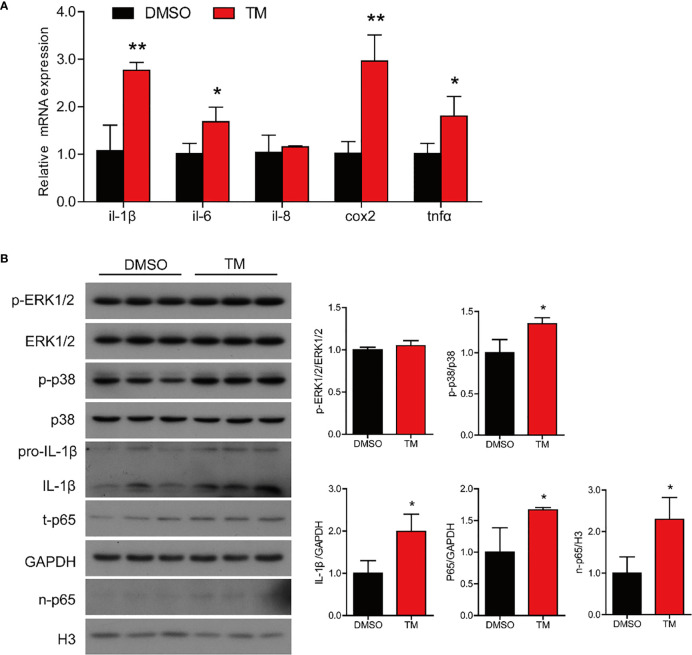
ER stress augments inflammatory response in the isolated intestinal cells of large yellow croaker *in vitro*. **(A)** Relative mRNA expression of inflammation-related genes in the isolated intestinal cells of large yellow croaker after TM treatment for 12 h (n = 3). **(B)** Western blot analysis of inflammation-related protein levels in the isolated intestinal cells of large yellow croaker after TM treatment for 12 h (normalized to GAPDH, n = 3). *il-1β*, interleukin-1 beta; *il-6*, interleukin-6; *il-8*, interleukin-8; *cox2*, cyclooxygenase 2; *tnfα*, tumor necrosis factor alpha; ERK1/2, extracellular signal-regulated protein kinases 1 and 2; p38, p38 mitogen-activated protein kinase; p65, NF-kappaB p65; GAPDH, glyceraldehyde-3-phosphate dehydrogenase. Results were analyzed using independent *t*-test (*P < 0.05, **P < 0.01), and they were presented as mean ± standard deviation.

We next examined the effect of ER stress on inflammatory response in the intestinal cells. TM treatment significantly upregulated the mRNA expression of proinflammatory genes, including *il-1β*, *il-6*, *cox2*, and *tnfα* (*P* < 0.05) (**Figure 7A**). Western blot results showed that TM treatment significantly increased the protein levels of p-p38/p38, total p65, IL-1β, and nuclear p65 (*P* < 0.05), while the protein level of p-ERK1/2/ERK1/2 was not remarkably different (*P* > 0.05) (**Figure 7B**). Collectively, these results indicated that ER stress induced inflammation in the isolated intestinal cells of large yellow croaker.

### UPR Sensors Regulate Promoter Activities of Proinflammatory Genes

**Figure 8:**
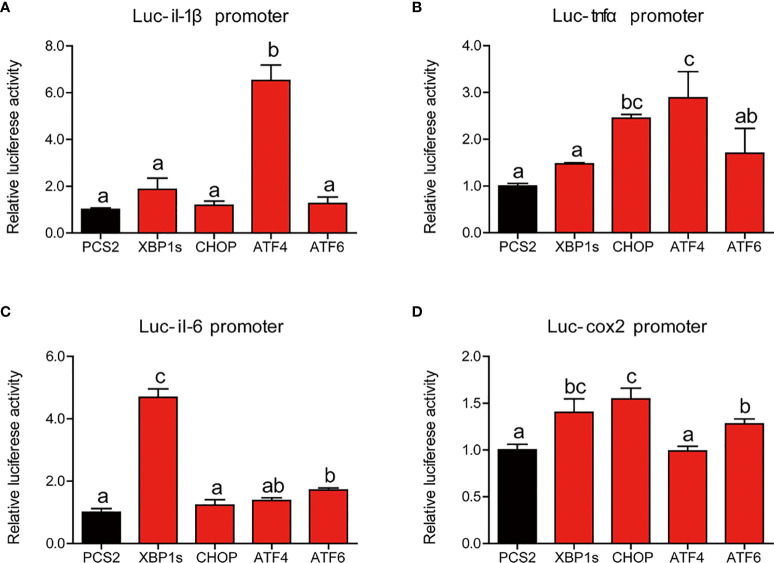
Relative dual-luciferase activity analysis of UPR sensors in promoters of proinflammatory genes in HEK293T cells. The promoter activities of *il-1β* **(A)**, *tnfα* **(B)**, *il-6* **(C)**, and *cox2* **(D)** were measured (n = 4). *il-1β*, interleukin-1 beta; *tnfα*, tumor necrosis factor alpha; *il-6*, interleukin-6; *cox2*, cyclooxygenase 2. Results were analyzed using Tukey’s test [values without the same letter indicate significant difference (*P* < 0.05)], and they were expressed as mean ± standard deviation.

Next, we further studied the mechanism of UPR sensors on the regulating transcriptional activity of proinflammatory genes. Compared with HEK293T cells transfected with PCS2+ plasmid, cells transfected with ATF4 expression plasmid showed significantly higher luciferase activity of the *il-1β* promoter of large yellow croaker (*P* < 0.05) (**Figure 8A**). Overexpression of CHOP or ATF4 expression plasmid significantly increased the promoter activity of *tnfα* compared with the control group (*P* < 0.05) (**Figure 8B**). Similarly, XBP1s or ATF6 could significantly upregulate the promoter activity of *il-6* (*P* < 0.05) (**Figure 8C**). Compared with the control group, HEK293 cells transfected with XBP1s, CHOP, or ATF6 expression plasmid showed significantly high promoter activity of *cox2* (*P* < 0.05) (**Figure 8D**).

## Discussion

The ER is the primary site for synthesizing and exporting proteins and lipids in cells (27). The ER homeostasis plays a critical role to maintain physiological function in response to extracellular changes (28). However, excessive accumulation of misfolded or unfold proteins in the ER leads to ER stress (29). Previous studies have demonstrated the effect of ER stress on lipid homeostasis and inflammation in the liver, related to several human liver diseases such as alcoholic fatty liver and non-alcoholic fatty liver (30–32). In fish, Cao et al. indicated that high fat diet blocked hepatic very-low-density lipoprotein secretion by activating the ER stress-associated IRE1/XBP1 pathway in blunt snout bream (*Megalobrama amblycephala*) (33). In yellow catfish (*Pelteobagrus fulvidraco*), the ER stress pathway played an important role in high glucose-induced changes of lipid metabolism (34). Our previous study also found that palmitic acid-induced ER stress, and the IRE1α pathway participated in palmitic acid-induced inflammation in the primary hepatocytes of large yellow croaker (35). However, the studies on the intestine are scarce, especially in fish. In the present study, we found that TM injection induced ER stress and activated UPR pathways in the intestine of large yellow croaker *in vivo*. And we also established an ER stress model in the isolated intestinal cells of large yellow croaker *in vitro*. TM treatment significantly upregulated ER-stress related genes and protein expressions in cells. These results were similar with previous studies in mammals (36, 37).

ER stress is closely related to lipid metabolism. Interestingly, we found that ER stress induced by TM disturbed the intestinal lipid metabolism resulting in abnormal TG accumulation in the intestine. In terms of intracellular lipid synthesis, the gene levels of *srebp1c*, *scd1*, *dgat1*, and *dgat2* and the protein levels of SREBP1c and ACC were significantly increased after TM injection. These may be the major contributor to ER stress-induced TG accumulation in the intestine. These results are in agreement with a previous study that pharmacologic ER stress promoted *de novo* lipogenesis in human hepatoma cells (38). After lipid synthesis, the intestinal lipids are stored as lipid droplets in the cytosol or transported to ER and packaged into chylomicrons (2). The gene expression of *adrp* was also upregulated in the TM group. ADRP played an important role in lipid droplet formation (39). Fei et al. (40) have demonstrated that ER stress could stimulate lipid droplet formation in *Saccharomyces cerevisiae*. The lipid droplets may contribute to reducing intracellular fatty acid-induced toxicity. The gene and protein expression of chylomicron secretion and fatty acid β-oxidation were also significantly upregulated upon the condition of ER stress, which may be an adaptive mechanism to relieve excessive lipid deposition in the intestine. However, previous studies showed that ER stress inhibited fatty acid β-oxidation gene expression, contributing to the early development of steatosis in the liver (41, 42). Thus, there may be different responding strategies to ER stress in different organs. Then we stimulated the insolated intestinal cells of large yellow croaker with 1 μM TM *in vitro*. The results were almost consistent with those *in vivo*. TM treatment significantly increased lipid synthesis resulting in excessive TG accumulation in cells, while TM treatment also upregulated fatty acid β-oxidation. To further investigate the regulatory mechanism of ER stress on lipid synthesis in the intestine, we cotransfected the reporter plasmids of lipid synthesis related promoters and expression plasmids of UPR sensors into HEK293T cells. We found that the overexpression of UPR sensors could significantly increase promoter activities of lipid synthesis related genes. These results were consistent with previous studies that XBP1 could directly bind to the promoter regions of the *dgat2* and *scd1* genes (43, 44). Overall, these results suggested that ER stress promoted lipid synthesis leading to abnormal lipid accumulation in the intestine.

Continual exposure to dietary metabolites, toxins impeded, exogenous antigens, and gut microflora makes the intestine susceptible to invasion by exogenous stress, ultimately burdening the ER resulting in ER stress (45). Unresolved ER stress can be the primary cause of inflammation in the intestine (46, 47). In the present study, we found that ER stress induced by TM significantly promoted the mRNA expression of proinflammatory genes and the protein levels of mature IL-1β and nuclear p65 in the intestine of large yellow croaker, while the MAPK pathway was not remarkably different. These results were consistent with previous studies in mammals (48–50). Thus, we speculated that ER stress might promote p65 into the nucleus to increase the transcription levels of proinflammatory genes, resulting in augmenting inflammatory response in the intestine. In an *in vitro* experiment, the results were similar with those in *in vivo*. TM treatment also induced inflammation in the isolated intestinal cells by increasing the transcriptional levels of proinflammatory genes and the protein levels of mature IL-1β, total p65, and nuclear p65. To further investigate how ER stress mediated inflammatory gene expression, we cotransferred UPR sensor expression plasmids and reporter plasmids of inflammatory genes’ promoters into HEK293T cells. We found that the overexpression of UPR sensors could significantly upregulate the promoter activities of proinflammatory genes. Previous studies have demonstrated that there are three pathways of UPR involved in regulating immunity and inflammation (15, 51, 52). For example, *xbp1* deletion in intestinal epithelium cells induced ER stress leading to organ-specific inflammation (10). Inhibition of ATF6 could attenuate chemokine (C-X-C motif) ligand 1 (CXCL1) and TNFα expression (46). Therefore, we demonstrated that ER stress induced inflammatory response in the intestine through promoting p65 into the nucleus and directly upregulating promoter activities of proinflammatory genes.

In conclusion, we reported that ER stress disturbed intestinal lipid metabolism homeostasis by promoting lipid synthesis resulting in abnormal lipid accumulation in the intestine and augmented inflammatory response through promoting p65 into the nucleus and directly upregulating the promoter activities of proinflammatory genes in large yellow croaker. These results indicated that attenuating ER stress may be an effective therapeutic strategy for maintaining lipid and immune homeostasis in the intestine of fish and human beings.

## Data Availability Statement

The original contributions presented in the study are included in the article/supplementary material. Further inquiries can be directed to the corresponding author.

## Ethics Statement

The animal study was reviewed and approved by the Institutional Animal Care and Use Committee of Ocean University of China. Written informed consent was obtained from the owners for the participation of their animals in this study.

## Author Contributions

WF and QA designed the experiments, performed the main experiments, and wrote the manuscript. QC and YS conducted other experiments. JL and ZZ analyzed and interpreted the data. YL and KM revised the manuscript. All authors contributed to the final editing and approval of the manuscript.

## Funding

This research is supported by the Key Program of National Natural Science Foundation of China (grant no: 31830103), the National Science Fund for Distinguished Young Scholars of China (grant no: 31525024), the Ten-thousand Talents Program (grant no: 2018-29), the Scientific and Technological Innovation of Blue Granary (grant no: 2018YFD0900402), and the Agriculture Research System of China (grant no: CARS-47-11).

## Conflict of Interest

The authors declare that the research was conducted in the absence of any commercial or financial relationships that could be construed as a potential conflict of interest.

## Publisher’s Note

All claims expressed in this article are solely those of the authors and do not necessarily represent those of their affiliated organizations, or those of the publisher, the editors and the reviewers. Any product that may be evaluated in this article, or claim that may be made by its manufacturer, is not guaranteed or endorsed by the publisher.
